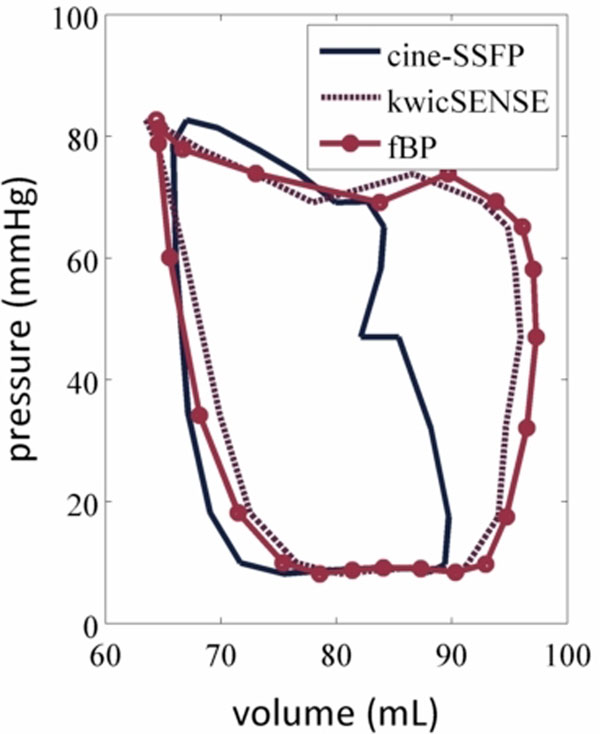# Real time measurement of cardiac pressure-volume relationships

**DOI:** 10.1186/1532-429X-14-S1-P227

**Published:** 2012-02-01

**Authors:** Walter R Witschey, Francisco J Contijoch, James J Pilla, Lawrence Dougherty, Hee Kwon Song, Melissa M Levack, Jeremy R McGarvey, Norihiro Kondo, Gerald A Zsido, Joseph H Gorman, Robert C Gorman

**Affiliations:** 1 Surgery, University of Pennsylvania, Glenolden, PA, USA; 2 Radiology, University of Pennsylvania, Philadelphia, PA, USA; 3 Bioengineering, University of Pennsylvania, Philadelphia, PA, USA

## Background

Clinical and experimental observations of pre- and afterload independent indices of impaired cardiac contractile performance and hemodynamic dysfunction are limited by the poor true temporal resolution of cine MRI. We aimed to develop and optimize for the first time an imaging and reconstruction protocol for MRI real time measurement of cardiac pressure-volume relations and to compare the results with cine-bSSFP.

## Methods

An MR imaging protocol was undertaken consisting of multislice, short-axis, cine-bSSFP and golden angle radial MRI performed on a clinical 3 T MRI scanner (Tim Trio Model, Siemens Healthcare) on 60 kg swine. A custom bSSFP, golden angle radial acquisition was run with a TR = 2.5 ms. Each Nth radial spoke was oriented along an azimuthal angle through the following schedule θ_i=N_i G, where the golden angle G=((3-√5))/2π. Coil sensitivity maps were obtained immediately following using an SPGR sequence and derived from image data using an adaptive coil combine. Two types of image reconstruction were performed: sum-of-squares filtered backprojection (fBP) and kwic-filtered iterative SENSE (kwicSense) using custom software on a GPU workstation. LV pressure was obtained from a transducer (Millar catheter) placed in the LV cavity. Cardiac volumes were segmented from cine-SSFP images in ITK-Snap and the slice-to-volume ratio was determined to estimate real time cardiac volumes.

## Results

Both fBP and kwicSense were found to increase the overall stroke volume by 5-6 mL compared to cine-SSFP acquisitions during free-breathing resting state conditions. The measured LVESV were similar for the three methods: 63.4 mL (kwicSense), 64.4 mL (fBP) and 65.9 mL (cine-bSSFP), although LVEDV was greater for the real time methods: 91.3 mL (kwicSense), 92.9 (fBP) and 89.4 (cine-SSFP). Small variations between segmentation of the volumes resulted in a left or right shift of the PV loop, although the shape of the loop, and consequently total myocardial work, was consistent. Blurring associated with the fBP method resulted in a greater myocardial signal intensity deteriorating the quality of image segmentation. Variations in the stroke volume may be related to changes in preload between cine-SSFP and real time acquisitions in the anesthetized animal.

## Conclusions

Real time MRI methods show great potential to characterize pre- and afterload independent indices of reduced cardiac contractile performance and hemodynamic dysfunction.

## Funding

The authors would like to thank Felix Wehrli for his generous support and encouragement and NIH grants T32-EB000814, R01-HL103723, R01-HL63954, R01-HL73021, and T32-EB009384.

**Figure 1:**